# Multiplex methylation-specific PCR for distinguishing semen, saliva, and blood

**DOI:** 10.1007/s00414-026-03803-3

**Published:** 2026-04-21

**Authors:** Kyeong-Min Yu, Eu-Ree Ahn, Myung Jin Park, Hyun-Chul Park

**Affiliations:** Forensic DNA Division, National Forensic Service, Wonju, 26460 Republic of Korea

**Keywords:** Methylation-specific PCR, Body fluid identification, Methylation, CpG site, Multiplex methylated PCR primer

## Abstract

Analysis of body fluids obtained from crime scenes provides important information for directing criminal investigations. Generally, chemical and immunological techniques, along with molecular methods that utilize RNA, are used for body fluid identification. However, these methods have some limitations, such as handling difficulty, sample consumption, false-positive or -negative results, and sample instability. To address these problems, several researchers have utilized DNA methylation for body fluid identification, including microarrays, combined bisulfite restriction analysis, single-base extension, and pyrosequencing. Although these methods have the advantage of simultaneously analyzing multiple target sites at the same time, they require considerable time and cost. In this study, a methylation-specific PCR (MSP)-based method was used to simultaneously discriminate between three human body fluids (semen, saliva, and blood). We designed primers with different amplicon sizes for each specific CpG region in the three body fluids (*ASIC4* for semen, *FAM43A* for saliva, and *FOXO3* for blood). For the accurate analysis, methylation and unmethylation primers were used separately for different amplifications. This MSP-based method can discriminate body fluids in unknown samples, although caution is required when analyzing mixed or low-quantity DNA samples. Our multiplex MSP-based approach enables confirmation of human body fluids and may provide a potentially simpler workflow compatible with conventional capillary electrophoresis platforms compared with methods such as the SNaPshot assay. We anticipate that this multiplex PCR system will be helpful in criminal investigations.

**Supplementary Information:**

The online version contains supplementary material available at 10.1007/s00414-026-03803-3.

## Introduction

Biological evidence detected at crime scenes plays an important role in case investigations [1, 2]. Body fluid identification aids in reconstructing the sequence of events at crime scenes. Generally, tests for discriminating human body fluids include chemical, enzymatic, or immunological assays. For example, luminol and leucomalachite green are chemical assays used for blood detection [3]. Phadebas® and SALIgAE® are also chemical tests to detect saliva in forensics [4]. Seminal acid phosphatase and prostate-specific antigen tests are useful chemical and immunological methods for semen detection [5]. These assays facilitate the straightforward identification of body fluids; however, some tests have several limitations such as sample consumption and their low sensitivity that can lead to false-positive or -negative results. Other assays that leverage differentially expressed mRNA levels between tissues have also been developed. However, they have limitations attributed to the greater instability of RNA compared with that of DNA and the need for careful handling to prevent degradation by ubiquitous RNases [6–10]. To address these issues, investigators have explored more accurate body fluid identification methods by leveraging epigenetics [11–13].

Epigenetics refers to various structural changes in a genome without alterations in the DNA sequence. Epigenetic modifications include histone modifications, DNA methylation, noncoding RNA action, and chromatin remodeling [14]. Among them, DNA methylation, a process in which a methyl group is added to the 5′ carbon of the cytosine within the pyrimidine base, regulates gene expression. Most methylation occurs at CpG sites, with 70–80% of these sites methylated in the human genome [2, 14–18]. DNA methylation controls mammalian development, including cell differentiation, embryonic development, X chromosome inactivation, genomic imprinting, and the inactivation of tumor suppressor genes [19, 20].

The identification of body fluids can be achieved by analyzing tissue-specific differentially methylated regions [21, 22]. The methods for analyzing methylation are diverse and include bead-based microarrays, combined bisulfite restriction analysis, single-base extension, pyrosequencing, and methylation-specific PCR (MSP). Herman et al. (1996) devised MSP, a simple and powerful method to confirm a specific methylation region in bisulfite-converted DNA using PCR [23]. Bisulfite treatment converts unmethylated cytosines to uracil, whereas methylated cytosines remain unchanged. During PCR, uracils are amplified to thymine, allowing the distinction between methylated and unmethylated cytosines. The MSP method requires methylated (M) and unmethylated (U) primers targeting specific CpG sites, and PCR amplification products are analyzed using gel electrophoresis to determine methylation status. Additionally, samples can be sensitively analyzed using capillary electrophoresis (CE) by attaching fluorescent tags to primers. Because of its simplicity, MSP is widely used to diagnose diseases associated with DNA methylation [24–26].

In this study, we distinguished three human body fluids: semen, saliva, and blood, by applying a multiplex MSP system. We designed M and U primers targeting the specific hypermethylated CpG sites in each body fluid sample (*ASIC4* for semen, *FAM43A* for saliva, and *FOXO3* for blood) based on the previous published papers [27, 28]. The sizes of the amplicons differed for each body fluid. Successful identification of all three body fluids in a single reaction was accomplished using case and mixed test samples. Furthermore, data analysis using CE instruments offers a more convenient, straightforward, and expedited alternative to traditional approaches.

## Materials and methods

### Marker selection and multiplex primer design

Target CpG sites with a high β-score, indicating a high methylation percentage, in the Illumina Human Methylation 450 K bead array, based on a previous study, were selected for the three body fluids (Table 1). The β-scores for the CpG sites targeting semen (cg17610929), blood (cg08792630), and saliva (cg09652652) were 0.92 ± 0.06, 0.50 ± 0.06, and 0.49 ± 0.17, respectively [27, 28]. Primers were designed to target CpG sites showing clear differential methylation between target and non-target body fluids, to be compatible with bisulfite-converted DNA, and to produce amplicons of distinct sizes suitable for multiplex capillary electrophoresis. Two sets of primers (M and U primers) were developed for the two separate amplifications. The 3' end of reverse primers was designed to be complementary to the CpG sites of each marker. The amplicons derived from semen, saliva, and blood using the M primers were the 71 bp, 95 bp, and 145 bp in length, respectively. Only forward primers were labeled with FAM dye at 5′ end, and the PCR products were distinguished based on their amplicon sizes during capillary electrophoresis analysis (Table 2). Degenerate bases were introduced at non-target CpG positions to accommodate sequence ambiguity resulting from bisulfite-conversion, where the methylation status of flanking CpGs is unknown. This approach was used to prevent preferential amplification of methylated or unmethylated alleles, while the target CpG site for discrimination was kept non-degenerate.

**Table 1:** Target CpG site information for body fluid identification

Target body fluid	Gene symbol	Target ID	CpG sites (hg 19)	Mean β-scores ± SD				References
				**Semen**	**Saliva**	**Blood**	**Vaginal fluid**	
Semen	*ASIC4*	cg17610929	Chr2:220,379,044	0.95 ± 0.06	0.01 ± 0.01	0.01 ± 0.01	0.01 ± 0.01	[27]
Saliva	*FAM43A*	cg09652652	Chr3:194,408,845	0.02 ± 0.01	0.49 ± 0.17	0.02 ± 0.00	0.03 ± 0.00	[27]
Blood	*FOXO3*	cg08792630	Chr6:108,883,909	0.08 ± 0.02	0.09 ± 0.01	0.50 ± 0.06	0.10 ± 0.02	[28]

**Table 2:** Primer information for multiplex methylation-specific PCR

Target body fluid	Primer sequence (5′–3′)^a, b^		Conc. (μM)	Amplicon size (bp)
Semen	M	F: GTTTTTGAYGTTYGTGTTGTYG	0.3	71
		R: GAAACCCTCCCCACG		
	U	F: GTTTGGTTTTTGATGTTTGTGTTGT	0.15	76
		R: AAAACCCTCCCCACA		
Saliva	M	F: TTTYGTTAGGTYGAYGGY	1.2	95
		R: CCACGAATAAATAACCACGATAAAACG		
	U	F: ATTTGTTTTGTTAGGTTGATGGTGT	0.09	100
		R: CCACAAATAAATAACCACAATAAAACA		
Blood	M	F: GGGTTTTGGTGTGGG	0.24	145
		R: CAAAAAAATAATAAAAAACGATAAAAAATCTCTCTTCG		
	U	F: GGGATGTGGGGTTTG	0.3	131
		R: CAAAAAAATAATAAAAAACAATAAAAAATCTCTCTTCA		

^a^F and R represent forward and reverse primers, respectively. M and U indicate methylated and unmethylated primers, respectively. The CpG sites are underlined

^b^ Mixed base: Y = T/C

### Sample collection

Sixty-seven body fluid samples (ten semen, thirty saliva, twenty-seven blood) were collected from healthy Korean 15 male and 15 female volunteers using analytical procedures approved by the Institutional Review Board of the National Forensic Service. All volunteers were informed about the goal and procedure of this study. Volunteers were asked to collect saliva into a 50-mL conical tube, and male volunteers were also asked fresh semen into a collection cup. Whole blood samples were collected using a syringe and used without the addition of any anticoagulants or preservatives (e.g., EDTA or citrate). All collected samples were aliquoted into microcentrifuge tubes of 200 μL each and stored at—20 °C before the following experiment. For the collection of vaginal fluid, cotton swabs were used, and it stored at room temperature in dry conditions. Amplified human genomic DNA (completely unmethylated), as well as completely methylated and unmethylated human control DNA (bisulfite-converted) from the EpiTect® PCR Control DNA kit (Qiagen, Hilden, Germany), were also used in this study. Presumptive testing was not repeated for the evidentiary samples that had been previously examined using the SM, LMG, and SALIgAE® tests, because these specimens were supplied as pre-screened forensic materials and additional chemical testing required more sample volume.

### DNA extraction and bisulfite-conversion

DNA was isolated from each sample using a QIAamp DNA Micro Kit (Qiagen). Quantification was conducted using a Quantifiler™ Trio DNA Quantification Kit (Thermo Fisher Scientific, Waltham, MA, USA) and a 7500 Real-Time PCR Instrument System (Thermo Fisher Scientific) according to the manufacturer's instructions. Bisulfite-conversion was performed using an EZ DNA Methylation-Lightning™ Kit (Zymo Research, Irvine, CA, USA) according to the manufacturer's instructions. To ensure consistency across samples, 20 ng of input DNA was used, with an elution volume of 10 μL. The bisulfite-converted DNA was stored at –80 °C until further use. In the mixture tests, all samples were used in the same quantity (20 ng) to prepare two- and three-sample mixtures. In addition, the input DNA for bisulfite-conversion was diluted two-fold for serial dilution tests from 20 to 1.25 ng.

### Methylation-specific PCR

Multiplex PCR amplification was conducted using an EpiScope® MSP kit (TAKARA Bio, Shiga, Japan) and a GeneAmp PCR System 9700 Thermal cycler (Applied Biosystems, Foster City, CA, USA) under the following conditions: initial activation at 95 °C for 30 s; 37 cycles of 98 °C for 5 s, 58 °C for 40 s, 60 °C for 40 s, and 72 °C for 1 min; and final extension at 72 °C for 20 min. Amplification was conducted using a 50-μL reaction volume comprising 5 μL of bisulfite-converted DNA, 25 μL of 2 × MSP Buffer, 0.5 μL of 100 × TB Green Solution, 1.2 μL of MSP enzyme, 18.3 μL of primers and distilled water. The optimized concentration for each primer is shown in Table 2. The PCR products were stored at 4 °C until the next procedure.

### CE and data analysis

The analysis included a 10-μL mixture of Hi-Di™ Formamide and GeneScan™ 600 LIZ™ dye Size Standard v2.0 (Applied Biosystems), along with 1 μL of PCR products. The mixtures were denatured at 95 °C for 3 min and cooled on a PCR cooler. Subsequently, the products were analyzed on a 3500 Genetic Analyzer (Applied Biosystems) using a 36-cm capillary array and POP-4™ Polymer (Applied Biosystems). The injection was conducted under the following conditions: 1.2 kV for 24 s, and electrophoresis was performed for 1,550 s at 13 kV and 60 °C. The data were analyzed using GeneMapper™ ID-X Software (Applied Biosystems).

### Set threshold of methylation level

The methylation level, defined as M/(M + U), was used to evaluate the methylation status, where M and U represent methylated and unmethylated RFUs, respectively. For each marker, a one-sided upper cutoff was defined from the distribution of negative (non-target) samples:

$$\text{cutoff }={\overline{x}}_{(neg)}+{{k}_{m}\cdot SD}_{(neg)}$$

Here, x denotes the methylation level M/(M + U); ${\overline{x}}_{(neg)}$ and ${{k}_{m}\cdot SD}_{(neg)}$ are the mean and standard deviation of x calculated from non-target (negative) samples. *k*_*m*_ is a marker-specific constant that controls the stringency of the one-sided cutoff for each target marker. A sample was classified as positive for the target body fluid when its methylation level was ≥ cutoff. To determine an appropriate constant *k*_*m*_ (*m* = [*ASIC4, FAM43A, FOXO3*]) for each target marker, we evaluated *k*_m_ = 1 to 5 using the negative (non-target) β-score distribution for each marker. We summarized the false-positive/false-negative trade-off across *k*_*m*_ values (Online Resource 1) and selected marker-specific *k*_*m*_ values to balance specificity and sensitivity. Based on this trade-off assessment, we used *k* = 5 for *ASIC4* and *FAM43A* to eliminate non-target false positives, and *k* = 2 for *FOXO3* to avoid excessive loss of blood sensitivity.

### Validation and performance metrics

To avoid optimism bias, cutoff estimation and performance evaluation were separated using leave-one-out cross-validation (LOOCV). In each LOOCV iteration, one single-source sample was held out as an independent test sample. The cutoff was calculated using only negative samples within the training set and then applied unchanged to the held-out sample. Predictions were aggregated over all iterations. Performance was evaluated in a one-vs-rest manner: *ASIC4* (semen vs saliva + blood), *FAM43A* (saliva vs semen + blood), and *FOXO3* (blood vs semen + saliva). Sensitivity, specificity, and accuracy were calculated from the aggregated confusion matrix and reported with 95% confidence intervals using Wilson binomial intervals. ROC curves and AUC were computed from continuous methylation levels.

### Composite decision rules for single-source and mixture calling

#### Composite decision rule for single-source and mixture inference (rule-based) with an inconclusive zone

For each marker, a one-sided upper cutoff was defined from the distribution of negative (non-target) samples:

$$\mathrm{c}={\overline{x}}_{(neg)}+{{k}_{m}\cdot SD}_{(neg)}$$

To reduce borderline calls, a marker-specific inconclusive (gray) zone was defined as [c-Δ, c + Δ], where Δ = ${SD}_{(neg)}$. Marker status was assigned as Positive if x *\ge* c + Δ, Negative if x *łe* c-Δ, and Inconclusive otherwise. Specimen-level interpretation used the following composite rule:

1. Single-source call: exactly one marker is Positive and the other two are Negative.

2. Mixture call: two or more markers are Positive (mixture composition reported as the set of Positive markers).

3. Inconclusive: any Inconclusive marker status, or no Positive markers.

Mixture performance was evaluated using replicate mixture experiments (three technical replicates per mixture type), and misclassification and inconclusive rates were summarized.

#### Composite-score classifier (LDA) with a probabilistic inconclusive rule

As an alternative composite rule, a linear discriminant analysis (LDA) classifier was trained using [x_(*ASIC4*)_, x_(*FAM43A*)_, x_(*FOXO3*)_] to classify seven classes (semen, saliva, blood, semen + saliva, semen + blood, saliva + blood, semen + saliva + blood). The predicted class was assigned as the class with the highest posterior probability, and samples were reported as Inconclusive if the maximum posterior probability was < 0.8. Model performance was evaluated using leave-one-out cross-validation (LOOCV) across all samples (single-source + mixture).

## Results and discussion

### Specificity tests

**Fig. 1:**
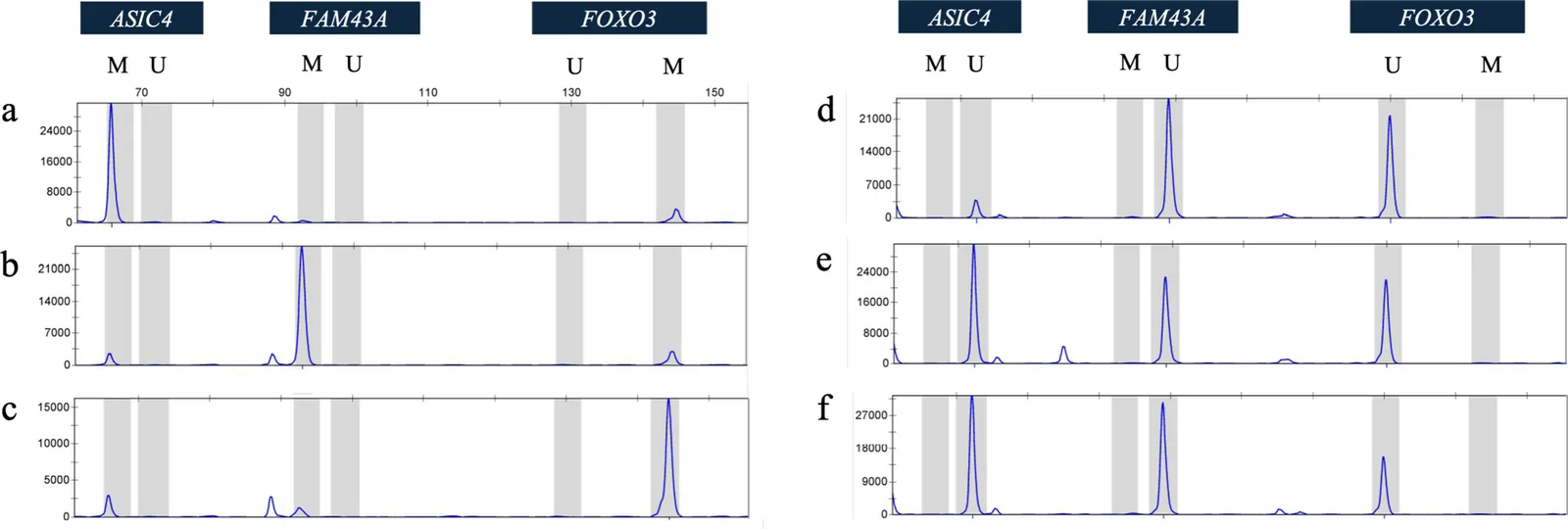
Electropherogram results of single body fluid samples amplified with tri-plex primer sets. Panels a and d show semen, panels b and e show saliva, and panels c and f show blood. M and U indicate methylated and unmethylated alleles, respectively

Sixty-seven body fluid samples were amplified using multiplex M and U primer sets. The selection of *ASIC4*, *FAM43A*, and *FOXO3* was guided by previous published studies [27, 28], which reported body fluid specific methylation markers for these loci, thereby reducing the potential for false classification in forensic applications. The representative samples from each body fluid are shown in Fig. 1. The M and U patterns of bisulfite-converted DNA from semen, saliva, and blood demonstrate successful amplification of the target markers. In semen, amplification with M primers yielded over 30,000 relative fluorescence units (RFUs) for the *ASIC4* marker (Fig. 1a). In contrast, amplification with U primers (Fig. 1d) in the semen samples produced the lowest RFU value for *ASIC4* (average = 3,119.6), whereas the average RFU values for *FAM43A* and *FOXO3* were 21,311.8 and 14,842.7, respectively. Saliva and blood samples amplified with M primers also exhibited the highest RFU values for each specific marker (over 15,000 RFU), but not as much as those for semen (Fig. 1b, c).

**Fig. 2:**
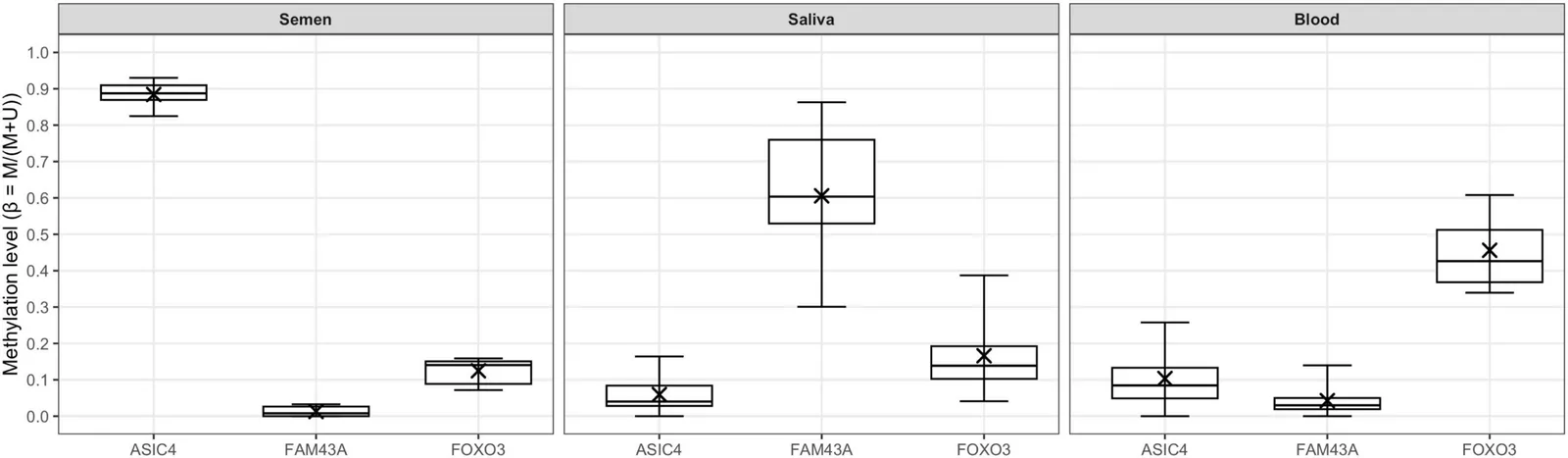
Box plots of methylation levels in semen, saliva, and blood samples. The methylation level for each marker was calculated as M/(M + U). The average methylation level of each box plot is indicated by an “x”

The methylation level, defined as M/(M + U), was used to evaluate the methylation status, where M and U represent methylated and unmethylated RFUs, respectively. As shown in Fig. 2, semen samples exhibited significantly higher methylation levels at the *ASIC4* marker compared to the other markers. Saliva and blood samples also displayed elevated methylation levels across their respective markers. However, all samples showed low methylation signals at the non-target markers (below 0.26), which may indicate cross-reactivity. Although we selected three markers with high average methylation percentage (β-scores) based on the study by Park et al. [28], these markers did not show 0% methylation in other body fluids. For the *ASIC4* gene, the β-score for blood and saliva was 0.10 ± 0.08 and 0.06 ± 0.05, respectively. In *FAM43A*, β-scores for semen and blood were 0.01 ± 0.01 and 0.04 ± 0.04, respectively. For the *FOXO3* gene, semen and saliva β-scores were 0.12 ± 0.04 and 0.17 ± 0.10, respectively. This indicates that methylation does not exhibit a strictly binary on/off pattern [28, 29]. The β-scores using the single-source samples in this study were calculated and presented in the Table 3. To reduce the risk of misinterpretation about false positives, the threshold values were determined as the ${\overline{x}}_{(neg)}+{{k}_{m}\cdot SD}_{(neg)}$.

**Table 3:** Mean β-scores for each marker in three body fluids, along with the threshold values. Bold values indicate the highest scores for each marker, while the highest non-specific values were underlined and used as thresholds

Marker name	Mean β-scores ± SD			Threshold : Mean β-scores + ${{k}_{m}\cdot SD}_{(neg)}$
	Semen (*n* = 10)	Saliva (*n* = 30)	Blood (*n* = 27)	
*ASIC4*	**0.88 ± 0.04**	0.06 ± 0.05	0.10 ± 0.08	0.431 (*k* = 5)
*FAM43A*	0.01 ± 0.01	**0.61 ± 0.19**	0.04 ± 0.04	0.237 (*k* = 5)
*FOXO3*	0.12 ± 0.04	0.17 ± 0.10	**0.46 ± 0.10**	0.341 (*k* = 2)

### Analytical specificity for the developed MSP multiplex assay

**Fig. 3:**
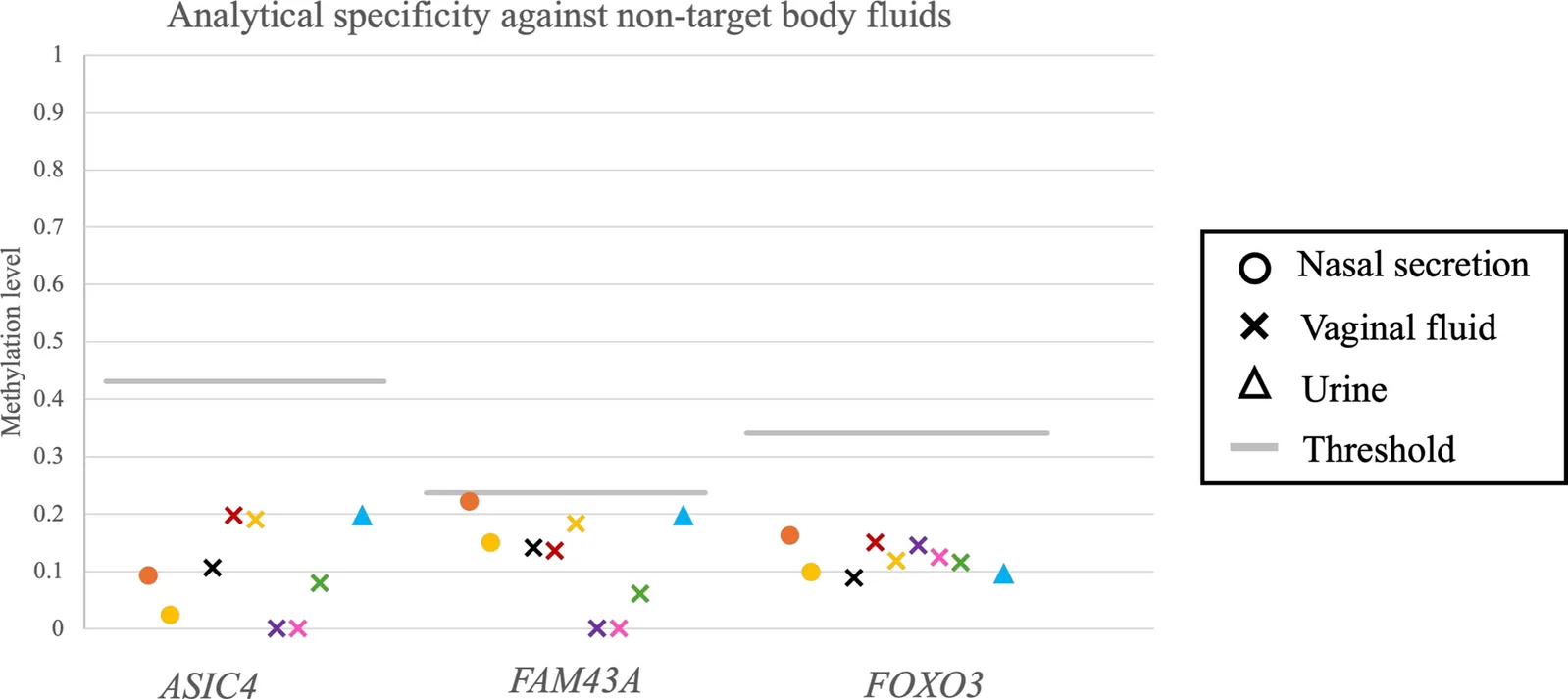
Methylation levels of each marker (*ASIC4*, *FAM43A*, and *FOXO3*) for six vaginal fluid, two nasal secretion, and one urine samples. Each symbol represents the following non-target body fluids: (●) Nasal secretion, (▲) Urine, and (✕) Vaginal fluid. The dashed line represents the established cut-off threshold

To evaluate the specificity of the developed MSP multiplex assay, cross-reactivity was assessed using nine non-target body fluids and genomic DNA from four non-human species. Among the non-target body fluids tested, vaginal fluid (*n* = 6), urine (*n* = 1), and nasal secretion (*n* = 2) all yielded beta score values below the established cut-off threshold, indicating no cross-reactivity with the target methylation markers (Fig. 3). These results confirm that the assay does not produce false-positive signals in the presence of these matrices. Sweat and skin/contact DNA samples were also subjected to the identical experimental procedure; however, no amplification was obtained following bisulfite conversion. This outcome is attributable to the inherently low DNA concentration characteristic of these sample types, which is a well-recognized limitation in forensic casework. As bisulfite treatment induces additional DNA degradation, the already limited template in sweat and skin/contact DNA was insufficient for downstream amplification. Nevertheless, this result is forensically relevant, as it indicates that these matrices would not generate false-positive interpretations when encountered as trace evidence at a crime scene.

For the assessment of species specificity, genomic DNA isolated from dog (*Canis lupus familiaris*), cow (*Bos taurus*), mouse (*Mus musculus*), and cat (*Felis catus*) was analyzed using the MSP multiplex assay. No amplification products were detected in any of the non-human DNA samples, demonstrating that the developed primers are strictly human-specific (Online Resource 2). This finding is particularly important in forensic investigations where biological evidence may be contaminated with or co-deposited alongside non-human biological material. Along with this, we also tested control DNA samples to confirm the absence of non-specific peaks for each marker; the data are shown in Online Resource 3. These results verify that our tri-plex primer sets correctly targeted semen, saliva, and blood samples, confirming their specificity. Collectively, these results confirm the high analytical specificity of the proposed MSP multiplex system, supporting its applicability in complex forensic scenarios involving mixed or potentially contaminated biological evidence.

### Sensitivity tests

**Fig. 4:**
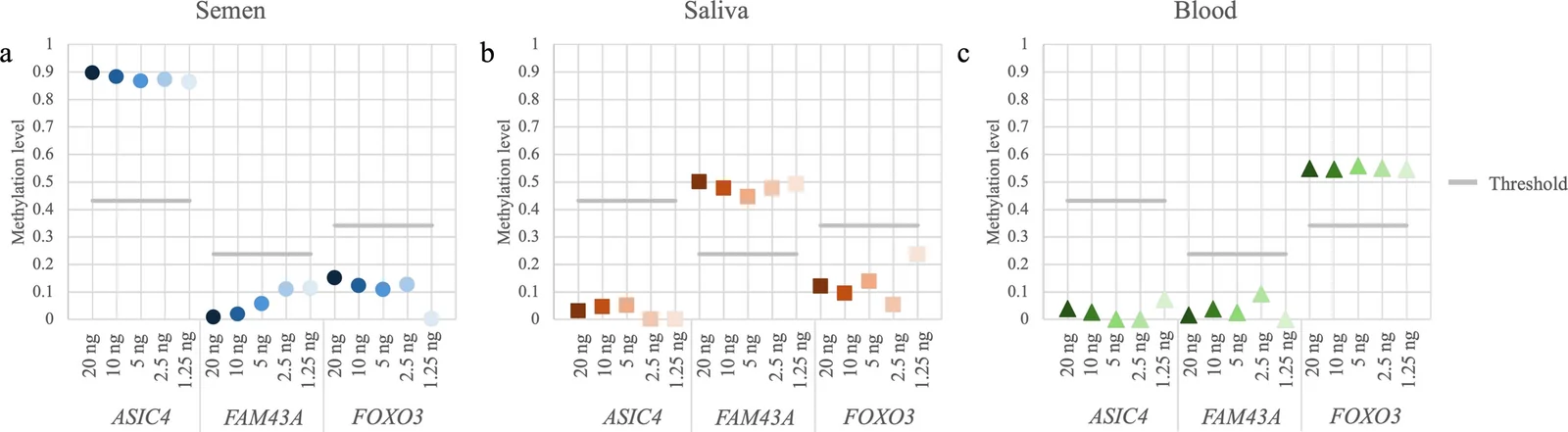
Methylation levels of each marker (*ASIC4*, *FAM43A*, and *FOXO3*) for serially diluted DNA samples from (a) semen, (b) saliva, and (c) blood using the DBB (Dilution Before Bisulfite-conversion) method. DNA input amounts ranged from 20 ng to 1.25 ng. The gray horizontal lines represent the threshold values for each marker

**Fig. 5:**
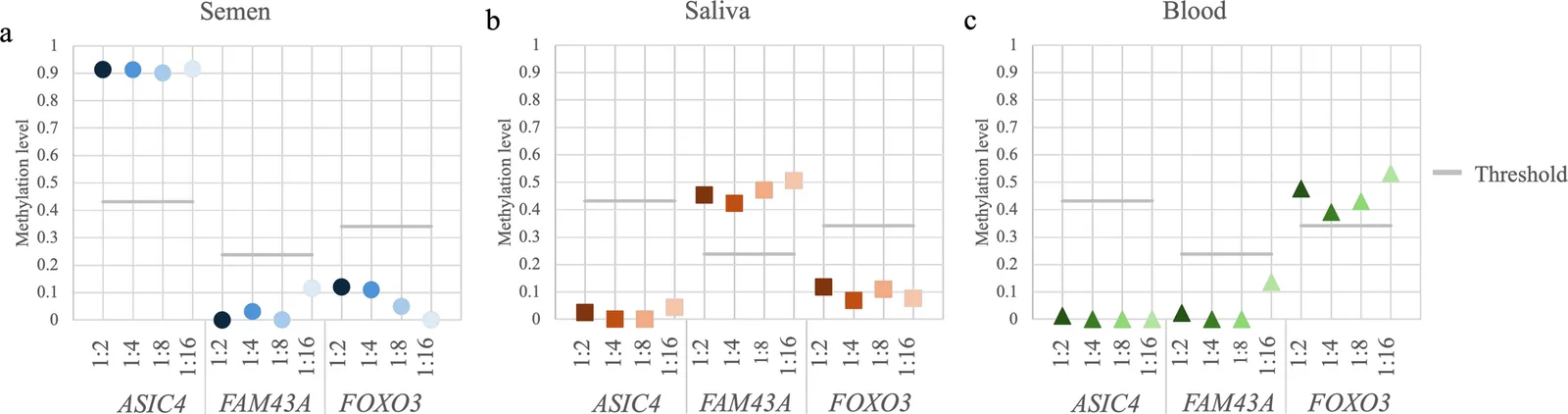
Methylation levels of each marker (*ASIC4*, *FAM43A*, and *FOXO3*) for serially diluted bisulfite-converted DNA from (a) semen, (b) saliva, and (c) blood using the DAB (Dilution After Bisulfite-conversion) method. Bisulfite-converted DNA prepared from 20 ng of input DNA was serially diluted two-fold up to 16-fold. Gray horizontal lines indicate the threshold values for each marker

Serial dilution experiments were conducted in each triplicate using two distinct approaches. First, DNA samples were diluted before bisulfite-conversion and subjected to bisulfite-conversion to assess the minimum DNA amount required for biological evidence in crime scene samples (Fig. 4). This approach is hereafter referred to as the Dilution Before Bisulfite-conversion (DBB) method. In the DBB method, the total DNA input was adjusted from 20 ng down to 1.25 ng. In the other approach, bisulfite-conversion was performed using 20 ng of DNA, and then the bisulfite-converted DNA was serially diluted two-fold to 16-fold. The diluted, bisulfite-converted DNA was subsequently used for PCR amplification (Fig. 5). This approach is hereafter referred to as the Dilution After Bisulfite-conversion (DAB) method. In the DBB method, almost of samples were fully detected using as little as 1.25 ng of DNA for conversion. The DAB method demonstrated that all body fluids could be detected even with a small amount of bisulfite-converted DNA (Fig. 5). The observed sensitivity is comparable to that reported in previous SNaPshot-based methylation studies [12, 27, 30], supporting the reliability of our method in producing results consistent with established standards.

### LOOCV validation of body fluid markers

Using LOOCV with training-only one-sided cutoffs (cutoff = ${\overline{x}}_{(neg)}+{{k}_{m}\cdot SD}_{(neg)}$), all three methylation markers showed strong discrimination for their target body fluids (Online resource 4). *ASIC4*, the semen-targeting marker, achieved perfect classification in this dataset with sensitivity 1.00 and specificity 1.00. *FAM43A*, the saliva-targeting marker, achieved sensitivity 0.967 with one false negative and specificity 1.000. *FOXO3*, the blood-targeting marker, achieved sensitivity 0.926 and specificity 0.950 with two false-positive non-target samples close to the cutoff. While the ROC curves for both *ASIC4* and *FAM43A* markers approached the upper-left corner and AUC values were 1.00, AUC value of *FOXO3* marker was shown by 0.973 (Online Resource 5), consistent with strong body-fluid specificity. Given the small sample size and near-complete separation, we emphasize binomial confidence intervals for sensitivity and specificity as primary uncertainty estimates.

### Mixed sample tests

**Fig. 6:**
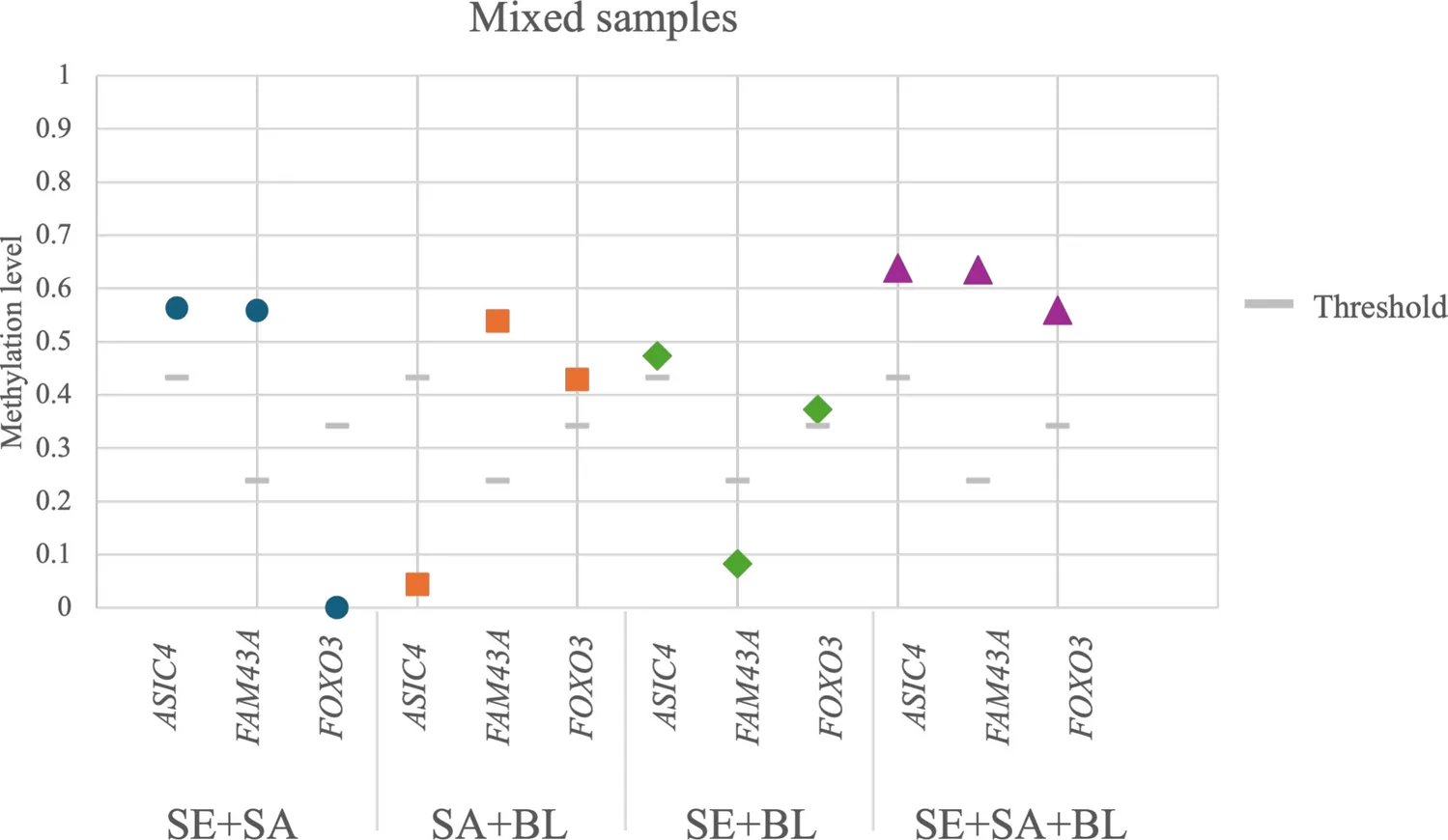
Methylation levels of each marker (*ASIC4*, *FAM43A*, and *FOXO3*) for mixed body fluid samples. Blue dots indicate mixtures of semen and saliva, orange squares indicate mixtures of saliva and blood, and green rhombuses indicate mixtures of semen and blood. The mixture of all three body fluids (semen, saliva, and blood) is shown in purple. Gray horizontal lines indicate the threshold values for each marker

We also prepared mixed samples by combining 20 ng of each DNA from two or three body fluids and used the mixtures in the bisulfite-conversion procedure. All the mixture tests were conducted in triplicate. The methylation levels in all mixtures showed good specificity, but the sensitivity differed slightly according to sample type (Fig. 6). As shown in the mixtures of saliva and blood, the methylation level for the *FAM43A* marker (0.539) was slightly different from that for the *FOXO3* marker (0.429). Also, in the mixtures of three body fluids, the methylation levels for the three markers were not displayed equally. Though we used the same DNA quantity, differences in the methylation status of each gene marker could affect this pattern. In this study, *ASIC4* in semen showed the highest average β-score (0.88), whereas *FAM43A* and *FOXO3* in saliva and blood exhibited lower scores (0.61 and 0.46)*.* In addition, the reduced detection rate observed for longer target sequences commonly suggests that amplicon size significantly could influence amplification efficiency [31, 32]. The intermediate methylation levels observed in mixture samples are an expected consequence of combining body fluids with distinct methylation profiles and do not indicate assay instability [33]. Furthermore, differential amplification efficiency may arise from unequal binding of primers to methylated versus unmethylated templates and the inclusion of CpG sites, leading to deviation from expected mean methylation ratios in mixture samples [34, 35]. As shown in Table 4, all mixed samples were correctly classified as mixtures, and none were misclassified as single-source samples. Although a subset of single-source samples was conservatively classified as mixtures, particularly in samples exhibiting borderline marker positivity. This method sacrifices single-source sensitivity to ensure zero false single-source calls from mixtures.

**Table 4:** Decision summary table for single-source and mixed samples. Samples were classified as single-source, mixture, or inconclusive based on multi-marker decision criteria

True status	Called as single	Called as mixture	Inconclusive
Single-source	65	2	0
Mixture	0	4	0

### Mixture inference under the predefined rule-based composite decision rule

Applying the predefined rule-based composite decision rule with an inconclusive zone to the mixture dataset (three technical replicates per mixture type) enabled explicit mixture calling and error quantification (Online Resource 6). Overall, 8/12 mixture replicates were correctly classified (66.7%), 2/12 were reported as inconclusive (16.7%), and 2/12 were misclassified (16.7%). Misclassifications were observed in semen + blood mixtures, where the saliva marker occasionally exceeded its threshold, leading to over-calling a three-fluid mixture.

### Composite-score model for combined single-source and mixture classification

The LDA composite-score model provided a multivariate alternative that formally integrates all three markers (Online Resource 7). Under LOOCV across all samples (single-source + mixture), the model achieved 76/79 correct classifications (96.2%) with one inconclusive calls at the prespecified posterior threshold. The single misclassification involved two saliva samples classified as a two-fluid mixture (Blood + Saliva), reflecting a trade-off between sensitivity to low-level components and conservative multivariate class boundaries.

### Performance of validation test from evidentiary samples

To assess the robustness of our MSP primer set, we evaluated its performance using DNA extracted from evidentiary samples collected from various crime scenes. A total of nine samples were analyzed using the same protocol as in the single-source test. It should be noted that the evidentiary samples used in this study had already undergone preliminary testing using the chemical or immunological methods during routine forensic examination, in which only one presumptive test was applied per sample based on the anticipated body fluid type. The results of the preliminary tests, MSP result interpretation, and the information about the nine evidentiary samples such as sample description and DNA concentration were shown in the Table 5.

**Table 5:** Information and results of evidence from real cases

Sample Name^a^	Sample Description	DNA Concentration (ng/μL)	Preliminary Test	MSP Result Interpretation^a^
SE1	Victim's vaginal fluid	101.3	SM positive	SE only
SE2	Stain on a bed	8.4	SM positive	SE only
SE3	Panties	1.6	SM positive	SE only
SA1	Swab collected from a drinking surface	1.6	SALIgAE® positive	SA only
SA2	cigarette butt	2.4	SALIgAE® positive	SA only
SA3	cigarette butt	5.7	SALIgAE® positive	SA only
BL1	Swab collected from blood stains on a tire	5	LMG positive	BL only
BL2	Swab collected from blood stains on the floor	1.1	LMG positive	BL only
BL3	Swab collected from kitchen knife	35.4	LMG positive	BL only

a. SE: semen, SA: saliva, and BL: blood.

**Fig. 7:**
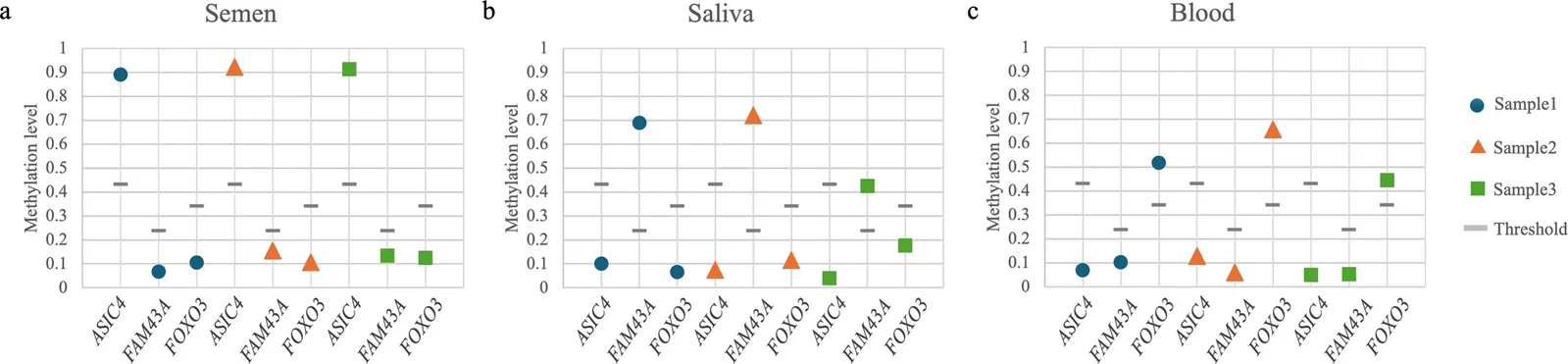
Methylation levels at each marker (*ASIC4*, *FAM43A*, and *FOXO3*) from the nine evidentiary samples. Panel a shows three semen samples. Panel b and c show three saliva and blood samples, respectively. Gray horizontal lines indicate the threshold values for each marker

The semen samples (SE1–SE3) demonstrated the most consistent performance, with successful detection. In the Fig. 7a, the methylation levels at the *ASIC4* marker were greater than 0.89 for all semen samples. In evidentiary samples presumed to originate from semen, differential lysis is typically performed to enrich sperm cell fractions by removing female epithelial cells. This process may increase the relative proportion of sperm-derived DNA, which can enhance the detectability of semen-associated methylation markers such as *ASIC4*.

The methylation levels detected in all saliva samples were above the predefined threshold, suggesting that the DNA most likely originated from saliva (Fig. 7b). In the Fig. 7c, blood markers exhibited methylation levels above 0.5 in the blood samples (BL1 and BL2), which were collected from visible bloodstains on surfaces using swabs. Sample BL3 obtained from the kitchen knife has a low methylation level as 0.44. Methylation-based interpretations should be considered within a broader forensic and biological context rather than as absolute indicators.

## Concluding remarks

Analysis of methylation at specific CpG sites can be useful for body fluid prediction in forensic studies. While several analytical methods, such as SNaPshot, chemical, and RNA-based assays, have been developed for body-fluid identification, some approaches may be associated with limitations, including the potential for false-positive or false-negative results, sample consumption, and the inherent instability of RNA. Therefore, we devised multiplex PCR primer sets that could simultaneously detect three types of body fluids (semen, saliva, and blood) using methylation-specific PCR. Three markers (*ASIC4*, *FAM43A*, and *FOXO3*), which have high β-scores for detecting specific body fluids, were selected based on previous research papers by Lee et al. [27] and Park et al. [28]. The methylation primers targeted only one type of body fluid; however, low RFU values of non-target markers were detected in all samples amplified with M primers. This phenomenon may be attributed to the β-scores for each body fluid. The methylation patterns were correctly represented in the mixture and control tests. Analyzing low amounts of DNA is challenging; however, the MSP method employed in this study provided reliable results even with limited DNA input, as evidenced by the serial dilution experiments. Furthermore, the MSP assay successfully identified the types of biological fluid in all mixed samples, demonstrating its potential to provide valuable investigative information regarding the circumstances of evidence deposition. In validation tests using evidentiary samples, the MSP primer set demonstrated the ability to discriminate potential sources of biological material, suggesting its potential applicability in forensic casework. The ability to detect and differentiate multiple biological fluids within a single-sample offers enhanced discriminatory power for forensic investigations, particularly when conventional methods yield ambiguous results. Nonetheless, forensic samples obtained from actual crime scenes may often contain substantially lower amounts of DNA than the inputs tested in this study. Therefore, further research is needed to develop more sensitive methods that can identify body fluids using minimal DNA. Furthermore, we aim to develop primer sets capable of distinguishing vaginal fluid and menstrual blood in sexual assault cases. This cutoff (${\overline{x}}_{(neg)}+{{k}_{m}\cdot SD}_{(neg)}$) was used as a conservative upper threshold to reduce false-positive calls by excluding the upper tail of the negative distribution, when specificity is prioritized in forensic decision-making.

## Supplementary Information

Below is the link to the electronic supplementary material.

Supplementary file1 (PDF 101 KB)

Supplementary file2 (PDF 864 KB)

Supplementary file3 (PDF 208 KB)

Supplementary file4 (PDF 133 KB)

Supplementary file5 (PDF 116 KB)

Supplementary file6 (PDF 108 KB)

Supplementary file7 (PDF 104 KB)

## Data Availability

The data supporting the findings of this study are available in the supplementary material.
