# Supplementary material for: Multiplex methylation-specific PCR for distinguishing semen, saliva, and blood
Source: Int J Legal Med. 2026 Apr 21;140(5):2607–17. doi: 10.1007/s00414-026-03803-3 (PMC13499774; doi:10.1007/s00414-026-03803-3)
Supplement: Supplementary file 1 [file 414_2026_3803_MOESM1_ESM.pdf]

Article title: Multiplex Methylation-specific PCR for Distinguishing Semen, Saliva, and Blood

Journal: International Journal of Legal Medicine

Authors: Kyeong-Min Yu, Eu-Ree Ahn, Myung Jin Park, Hyun-Chul Park

Affiliation: Forensic DNA Division, National Forensic Service

Corresponding author: Hyun-Chul Park

Email: hcpark79@korea.kr

Online Resource 1 Evaluation of k_m= 1 to 5 using the negative (non-target) β-score distribution for each marker.

Marker	Target	*k*	Cutoff	FP	FN
ASIC4	semen	1	0.150	7/57 (12%)	0/10 (0.0%)
ASIC4	semen	2	0.220	2/57 (3.5%)	0/10 (0.0%)
ASIC4	semen	3	0.291	1/57 (1.7%)	0/10 (0.0%)
ASIC4	semen	4	0.361	0/57 (0.0%)	0/10 (0.0%)
ASIC4	**semen**	**5**	**0.431**	**0/57 (0.0%)**	0/10 (0.0%)
FAM43A	saliva	1	0.075	5/37 (13.5%)	0/30 (0.0%)
FAM43A	saliva	2	0.115	3/37 (8.1%)	0/30 (0.0%)
FAM43A	saliva	3	0.156	1/37 (2.7%)	0/30 (0.0%)
FAM43A	saliva	4	0.196	0/37 (0.0)	0/30 (0.0%)
FAM43A	**saliva**	**5**	**0.237**	**0/37 (0.0)**	**1/30 (3.3%)**
FOXO3	blood	1	0.248	5/40 (12.5%)	0/27 (0.0%)
FOXO3	**blood**	**2**	**0.341**	**2/40 (5%)**	**2/27 (7.4%)**
FOXO3	blood	3	0.434	2/40 (5%)	14/27 (51%)
FOXO3	blood	4	0.527	0/40 (0.0%)	21/27 (77%)
FOXO3	blood	5	0.619	0/40 (0.0%)	25/27 (92.5%)

ASIC4: Positive (semen) 10, Negative (saliva+blood) 57

FAM43A: Positive (saliva) 30, Negative (semen+blood) 37

FOXO3: Positive (blood) 27, Negative (saliva+semen) 40
