# Supplementary material for: Multiplex methylation-specific PCR for distinguishing semen, saliva, and blood
Source: Int J Legal Med. 2026 Apr 21;140(5):2607–17. doi: 10.1007/s00414-026-03803-3 (PMC13499774; doi:10.1007/s00414-026-03803-3)
Supplement: Supplementary file 2 [file 414_2026_3803_MOESM2_ESM.pdf]

Article title: Multiplex Methylation-specific PCR for Distinguishing Semen, Saliva, and Blood

Journal: International Journal of Legal Medicine

Authors: Kyeong-Min Yu, Eu-Ree Ahn, Myung Jin Park, Hyun-Chul Park

Affiliation: Forensic DNA Division, National Forensic Service

Corresponding author: Hyun-Chul Park

Email: hcpark79@korea.kr

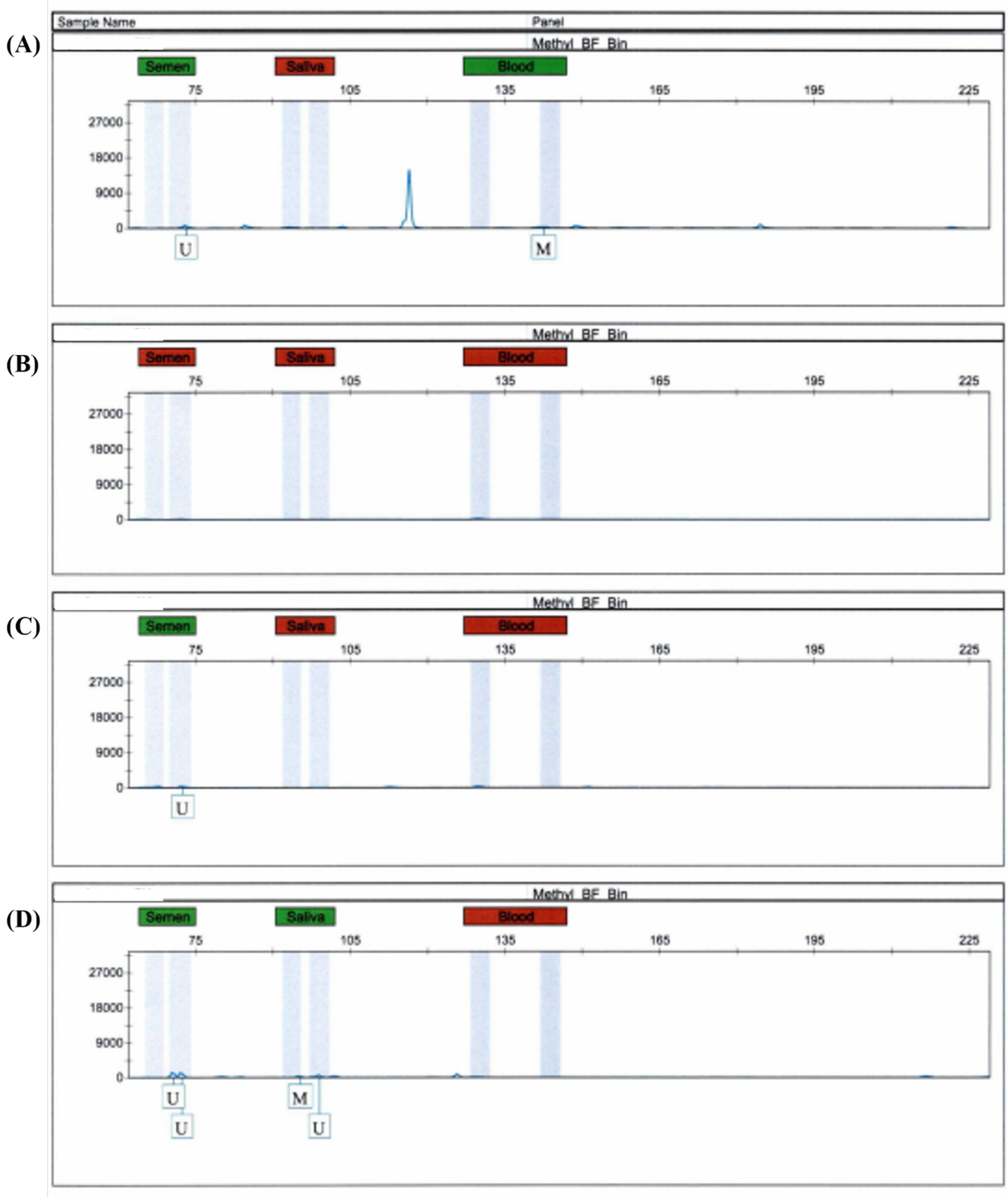

Online Resource 2 Electropherograms demonstrate the absence of amplification products in all non-human DNA samples tested. Genomic DNA from (a) dog (*Canis lupus familiaris*), (b) cow (*Bos taurus*), (c) mouse (*Mus musculus*), and (d) cat (*Felis catus*) was subjected to bisulfite conversion and MSP multiplex amplification. No peaks were detected within the designated marker bins for semen, saliva, or blood, confirming that the developed primers are strictly human-specific.
