# Supplementary material for: Multiplex methylation-specific PCR for distinguishing semen, saliva, and blood
Source: Int J Legal Med. 2026 Apr 21;140(5):2607–17. doi: 10.1007/s00414-026-03803-3 (PMC13499774; doi:10.1007/s00414-026-03803-3)
Supplement: Supplementary file 3 [file 414_2026_3803_MOESM3_ESM.pdf]

Article title: Multiplex Methylation-specific PCR for Distinguishing Semen, Saliva, and Blood

Journal: International Journal of Legal Medicine

Authors: Kyeong-Min Yu, Eu-Ree Ahn, Myung Jin Park, Hyun-Chul Park

Affiliation: Forensic DNA Division, National Forensic Service

Corresponding author: Hyun-Chul Park

Email: hcpark79@korea.kr

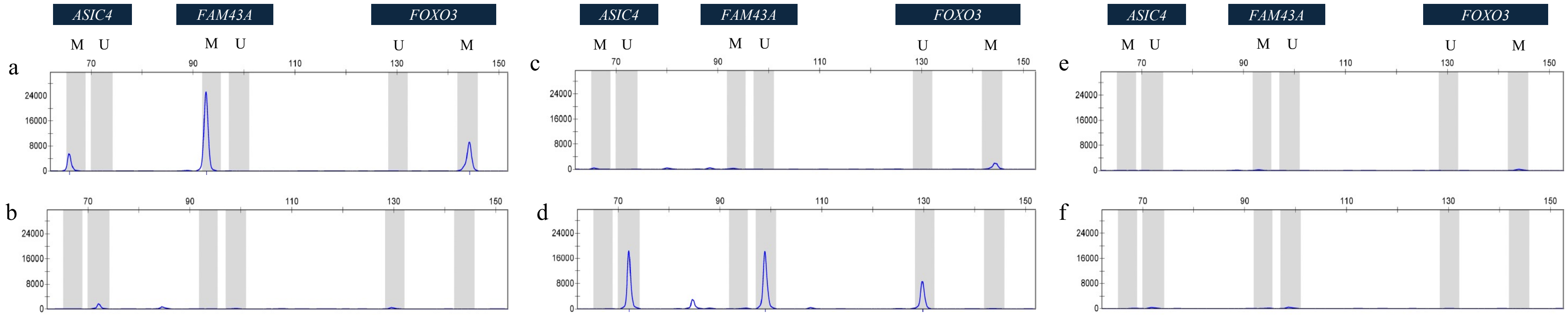

Online Resource 3 Electropherograms for control DNA amplified with tri-plex primer sets. Completely methylated human control DNA (converted; a and b), completely unmethylated human control DNA (converted; c and d), and amplified human genomic DNA (completely unmethylated; e and f) were used as the control DNA. Panels a, c, and e show the amplification with M primers. Panels b, d, and f show the amplification with U primers. M and U represent methylated and unmethylated
