# Supplementary material for: Multiplex methylation-specific PCR for distinguishing semen, saliva, and blood
Source: Int J Legal Med. 2026 Apr 21;140(5):2607–17. doi: 10.1007/s00414-026-03803-3 (PMC13499774; doi:10.1007/s00414-026-03803-3)
Supplement: Supplementary file 4 [file 414_2026_3803_MOESM4_ESM.pdf]

Article title: Multiplex Methylation-specific PCR for Distinguishing Semen, Saliva, and Blood

Journal: International Journal of Legal Medicine

Authors: Kyeong-Min Yu, Eu-Ree Ahn, Myung Jin Park, Hyun-Chul Park

Affiliation: Forensic DNA Division, National Forensic Service

Corresponding author: Hyun-Chul Park

Email: hcpark79@korea.kr

Online Resource 4 LOOCV performance for each methylation marker using training-only one-sided cutoffs ($\overline{x}_{(neg)} + k_m \cdot SD_{(neg)}$)

Marker	Poitive / Negative definition	*K*	Cut-off	Sensitivity (95% CI)	Specificity (95% CI)	Accuracy (95% CI)	ROC-AUC	TP/FN/TN/FP
ASIC4	SE (10) vs (SA+BL (57))	5	0.431	1.000(0.722-1.000)	1.000(0.937-1.000)	1.000(0.946-1.000)	1.00	10/0/57/0
FAM43A	SA (30) vs (SE+BL (37))	5	0.237	0.967(0.833-0.996)	1.000(0.906-1.000)	0.985(0.920-0.997)	1.00	29/1/37/0
FOXO3	BL (27) vs (SE+SA (40))	2	0.341	0.926(0.761-0.983)	0.950(0.832-0.986)	0.940(0.856-0.977)	0.973	25/2/38/2

SE: semen, SA: saliva, and BL: blood.

Methylation level was calculated as M/(M+U). In each LOOCV iteration, the cutoff was computed only from negative training samples as $\overline{x}_{(neg)} + k_m \cdot SD_{(neg)}$, and applied unchanged to the held-out test samples. Confidence intervals(CI) for sensitivity, specificity, and accuracy were calculated using Wilson binomial intervals.
