# Supplementary material for: Multiplex methylation-specific PCR for distinguishing semen, saliva, and blood
Source: Int J Legal Med. 2026 Apr 21;140(5):2607–17. doi: 10.1007/s00414-026-03803-3 (PMC13499774; doi:10.1007/s00414-026-03803-3)
Supplement: Supplementary file 5 [file 414_2026_3803_MOESM5_ESM.pdf]

Article title: Multiplex Methylation-specific PCR for Distinguishing Semen, Saliva, and Blood

Journal: International Journal of Legal Medicine

Authors: Kyeong-Min Yu, Eu-Ree Ahn, Myung Jin Park, Hyun-Chul Park

Affiliation: Forensic DNA Division, National Forensic Service

Corresponding author: Hyun-Chul Park

Email: hcpark79@korea.kr

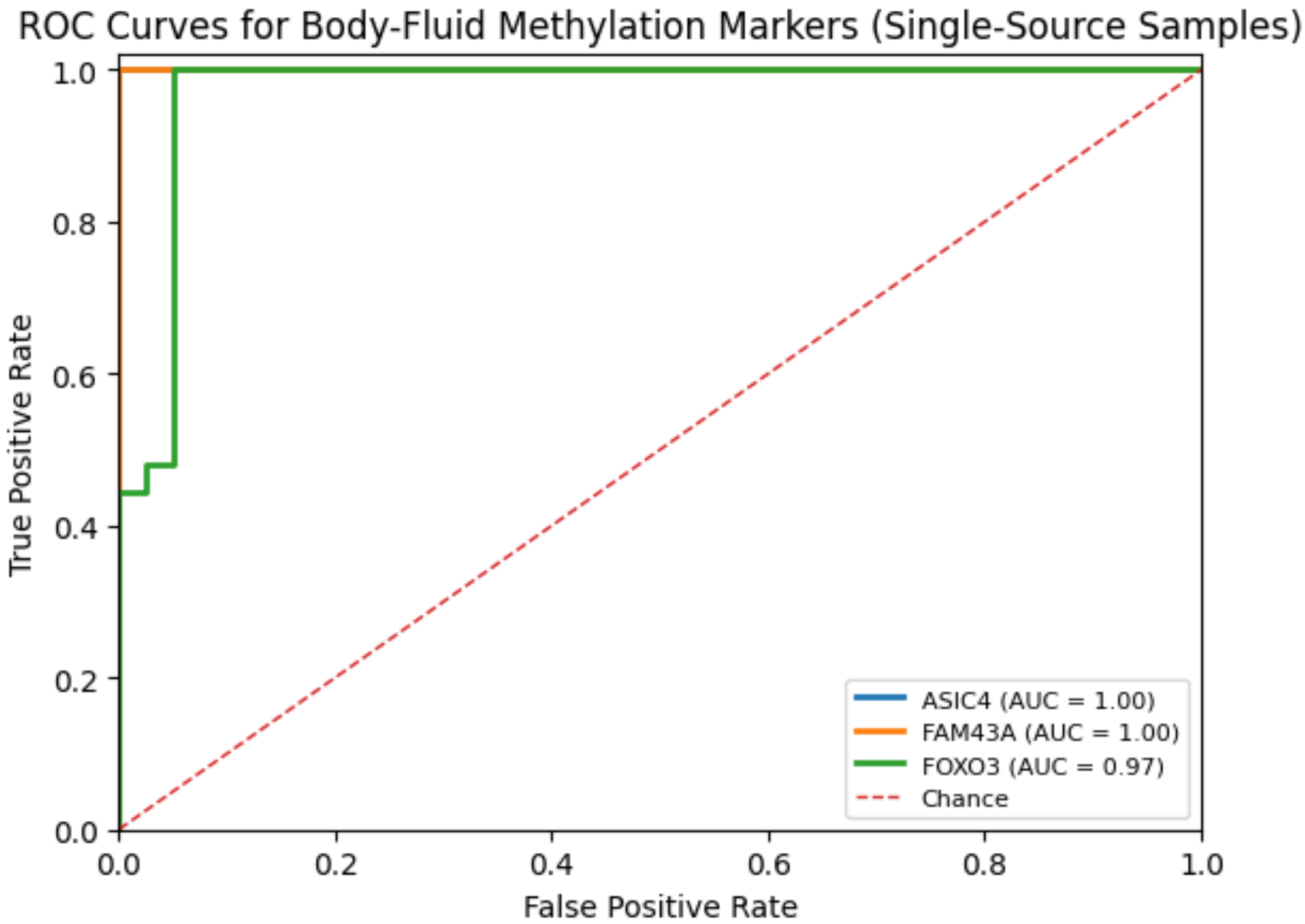

Online Resource 5 ROC curves for single-source body-fluid classification using methylation level.

ROC curves were generated using continuous methylation level (M/(M+U)) for each marker in a one-vs-rest manner (*ASIC4*: semen vs saliva+blood; *FAM43A*: saliva vs semen+blood; *FOXO3*: blood vs semen+saliva). The dashed diagonal indicates chance-level performance.
