# Supplementary material for: Multiplex methylation-specific PCR for distinguishing semen, saliva, and blood
Source: Int J Legal Med. 2026 Apr 21;140(5):2607–17. doi: 10.1007/s00414-026-03803-3 (PMC13499774; doi:10.1007/s00414-026-03803-3)
Supplement: Supplementary file 6 [file 414_2026_3803_MOESM6_ESM.pdf]

Article title: Multiplex Methylation-specific PCR for Distinguishing Semen, Saliva, and Blood

Journal: International Journal of Legal Medicine

Authors: Kyeong-Min Yu, Eu-Ree Ahn, Myung Jin Park, Hyun-Chul Park

Affiliation: Forensic DNA Division, National Forensic Service

Corresponding author: Hyun-Chul Park

Email: hcpark79@korea.kr

Online Resource 6 Performance of the rule-based composite decision rule on mixture samples using thresholds trained on single-source negatives.

True class	*n*	Correct	Inconclusive	Misclassified	Most common error (if any)
saliva+semen	3	2	1	0	–
blood+saliva	3	3	0	0	–
blood+semen	3	0	1	2	blood+saliva+semen
blood+saliva+semen	3	3	0	0	–
**Overall**	**12**	**8 (66.7%)**	**2 (16.7%)**	**2 (16.7%)**	**–**
