# Supplementary material for: Multiplex methylation-specific PCR for distinguishing semen, saliva, and blood
Source: Int J Legal Med. 2026 Apr 21;140(5):2607–17. doi: 10.1007/s00414-026-03803-3 (PMC13499774; doi:10.1007/s00414-026-03803-3)
Supplement: Supplementary file 7 [file 414_2026_3803_MOESM7_ESM.pdf]

Article title: Multiplex Methylation-specific PCR for Distinguishing Semen, Saliva, and Blood

Journal: International Journal of Legal Medicine

Authors: Kyeong-Min Yu, Eu-Ree Ahn, Myung Jin Park, Hyun-Chul Park

Affiliation: Forensic DNA Division, National Forensic Service

Corresponding author: Hyun-Chul Park

Email: hcpark79@korea.kr

Online Resource 7 LDA composite-score classifier performance using the three marker methylation levels (LOOCV).

True class	*n*	Correct	Misclassified	Inconclusive	Most common error (if any)
semen	10	10	0	0	–
saliva	30	28	2	0	Blood+Saliva
blood	27	27	0	0	–
saliva + semen	3	3	0	0	–
blood + saliva	3	3	0	0	–
blood + semen	3	3	0	0	–
blood + saliva + semen	3	2	0	1	inconclusive
**Overall**	**79**	**76 (96.2%)**	**2 (2.5%)**	**1 (1.3%)**	–
